# Supplementary material for: Steering Away from Current Amoxicillin Dose Reductions in Hospitalized Patients with Impaired Kidney Function to Avoid Subtherapeutic Drug Exposure
Source: Antibiotics (Basel). 2022 Sep 2;11(9):1190. doi: 10.3390/antibiotics11091190 (PMC9494977; doi:10.3390/antibiotics11091190)
Supplement: Supplementary file 1 [file antibiotics-11-01190-s001.zip › antibiotics-1817837-supplementary.pdf]

Supplementary

Title

Steering away from current amoxicillin dose reductions in hospitalized patients with impaired kidney function to avoid subtherapeutic drug exposure

Authors

Cornelis Smit*, Swapnoleena Sen*, Elodie von Dach, Abderrahim Karmime, Pierre Lescuyer, David Tonoli, Julia Bielicki, Angela Huttner, Marc Pfister

* shared first authorship

Content

Table S1	2
Table S2	2
Figure S1	3
Figure S2	4
Figure S3	5
Figure S4	6
Figure S5	7
Figure S6	8
Figure S7	9
Figure S8	10

Tables

Table S1. Results of implementing different kidney function estimates on amoxicillin CL

**Model**	**Parameter relationship**	**β_{cov} (RSE%)**	**OFV**	**ΔOFV**	**P-value (compared to reference)**
Structural PK model (allometric WT on CL/Vd)	-	-	1314.2	(reference)	-
CKD-EPI on CL	Exponential	1.20 (8.4)	1172.9	-141.3	<0.001
**CKD-EPI on CL**	**Linear**	**1 FIX**	**1174.3**	**-139.9**	**<0.001**
MDRD on CL	Exponential	1.06 (7.7)	1177.1	-137.1	<0.001
MDRD on CL	Linear	1 FIX	1176.7	-137.5	<0.001
CG on CL	Exponential	0.88 (8.6)	1209.7	-104.5	<0.001
CG on CL	Linear	1 FIX	1212.0	-102.3	<0.001

Table S2. Results of different capping values for CKD-EPI

**Model**	**Parameter relationship**	**β_{cov} (RSE%)**	**OFV**	**ΔOFV**	**P-value (compared to reference)**
CKD-EPI on CL	-	1.20 (8.4)	1172.9	(reference)	-
CKD-EPI (capped at 120 mL/min/1.73 m^2) on CL	Exponential	1.28 (8.5)	1182.2	+9.3	>0.05
CKD-EPI (capped at 130 mL/min/1.73 m^2) on CL	Exponential	1.26 (8.4)	1180.8	+7.9	>0.05
CKD-EPI (capped at 140 mL/min/1.73 m^2) on CL	Exponential	1.23 (7.9)	1174.5	+1.6	>0.05

Figures

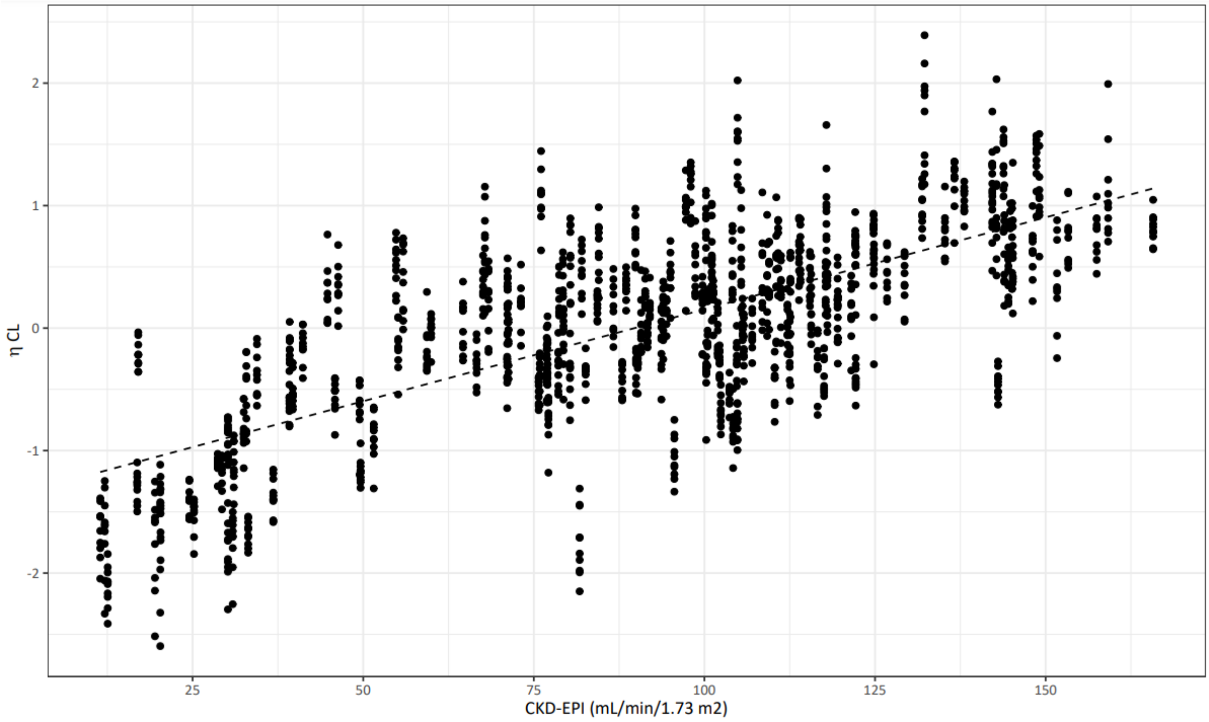

Figure S1. Inter-individual variability on CL estimates (ηCL), derived from the conditional distribution (n=7 repeats per individual) versus kidney function in CKD-EPI (in mL/min/1.73 m^2), based on the structural model. Dashed line shows regression line (r^2 = 0.71).

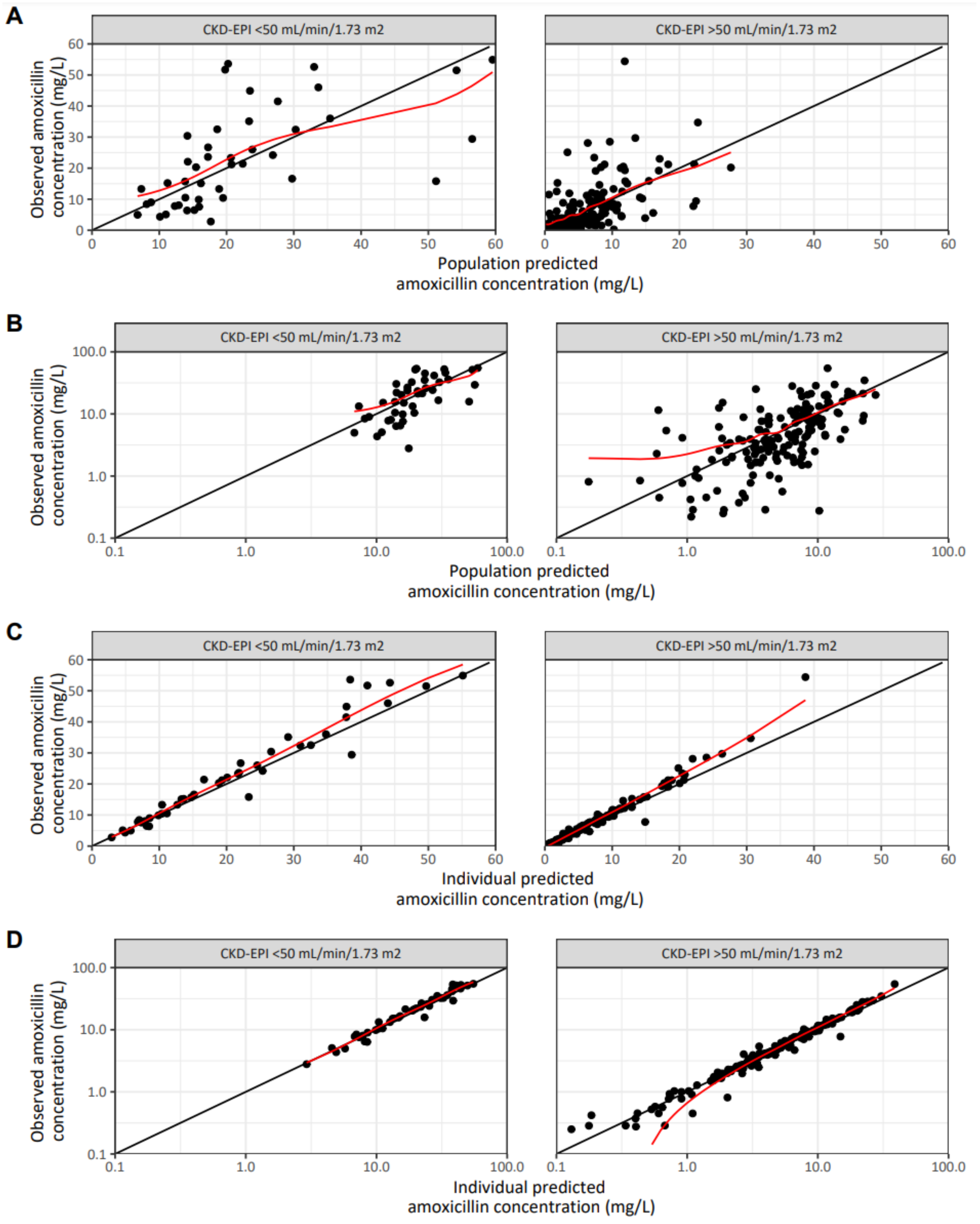

Figure S2. Goodness-of-fit plots for the final pharmacokinetic model. Shown are observed concentrations versus population predicted on normal (A) and logarithmic (B) scale and individual predicted concentrations on normal (C) and logarithmic (D) scale, split for kidney function (CKD-EPI) below and above 50 mL/min/1.73 m^2. The black circles correspond to an observed value, the black line is the y=x line and the red line depicts the spline interpolation.

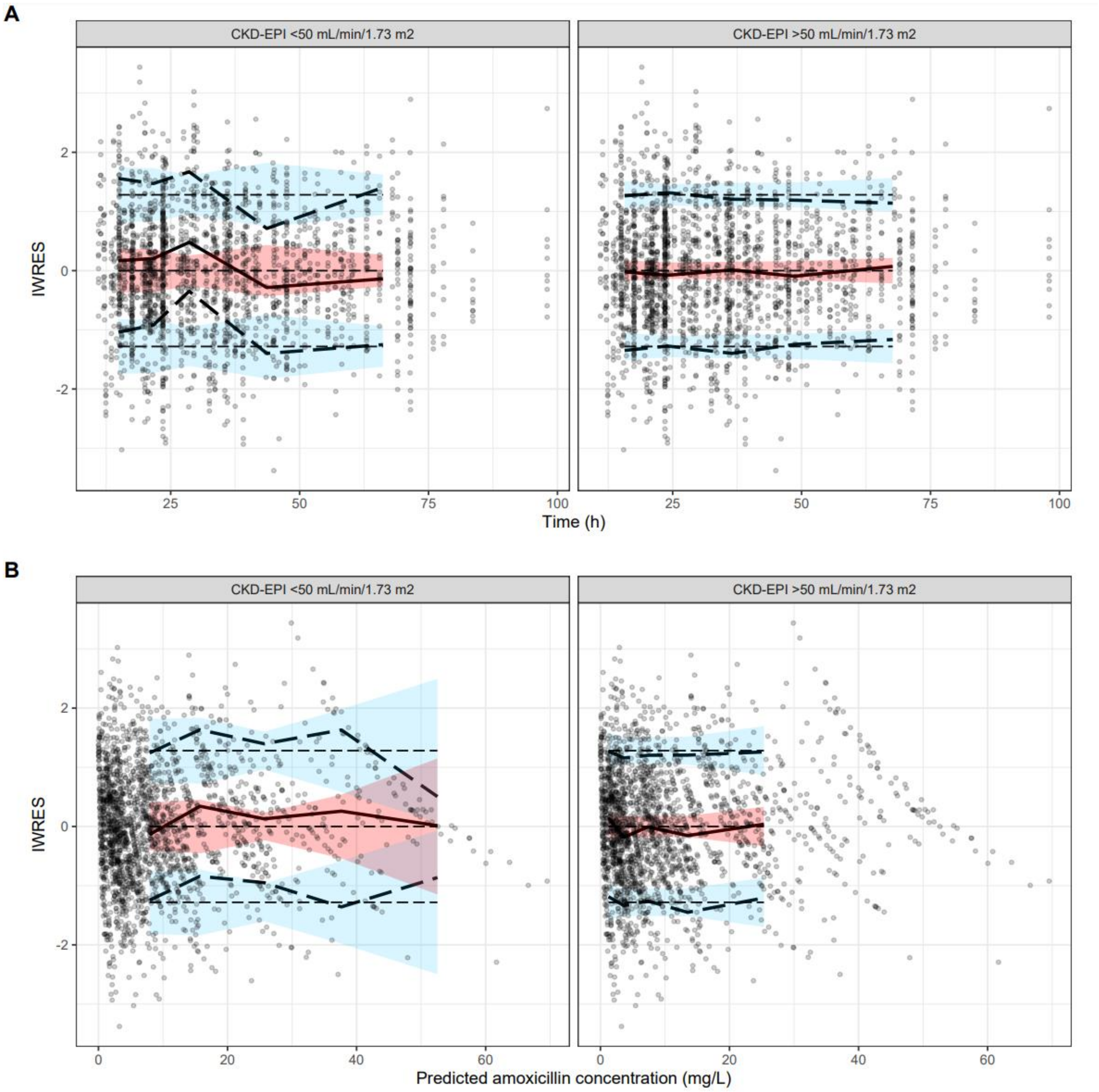

Figure S3. IWRES versus time (in hours, A) or predicted amoxicillin concentration (in mg/L, B), split for kidney function (CKD-EPI) below and above 50 mL/min/1.73 m^2. 50th, 5th and 95th percentiles of the predicted IWRES are shown in red and blue shaded areas, whereas observed values (derived from the conditional distribution, n = 7 repeats per individual) are depicted by the dots, with the solid and thick dashed lines representing the 50th, 5th and 95th percentiles of the observations respectively. Thin dashed lines show the theoretical mean and predicted percentiles. *IWRES* individual weighted residual.

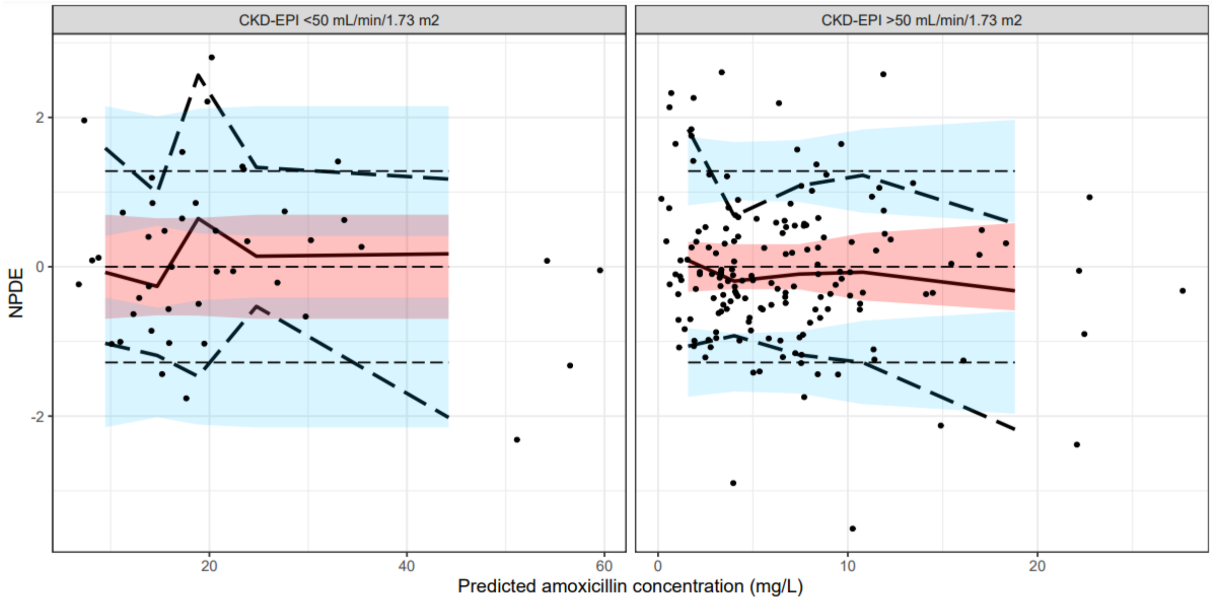

Figure S4. Observed and expected NPDE versus predicted amoxicillin concentration (in mg/L), split for kidney function (CKD-EPI) below and above 50 mL/min/1.73 m^2. 50th, 5th and 95th percentiles of the expected NPDE are shown in red and blue shaded areas, whereas observed values are depicted by the dots, with the solid and dashed lines representing the 50th, 5th and 95th percentiles of the observations respectively. Thin dashed lines show the theoretical mean and predicted percentiles. *NPDE* normalized prediction distribution error.

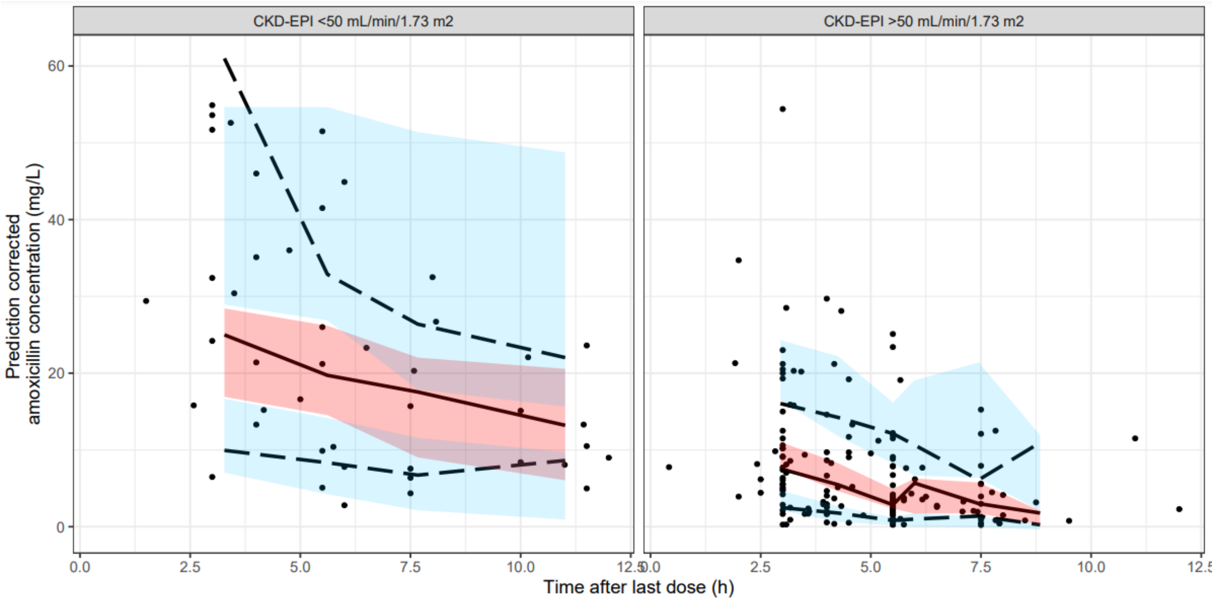

Figure S5. Prediction corrected Visual Predictive Check for the final model versus time after last dose (in hours), split for kidney function (CKD-EPI) below and above 50 mL/min/1.73 m^2. Lines represent the 5th (dashed), 50th (solid), and 95th (dashed) percentiles of the prediction corrected observed data. The shaded areas indicate the 90% confidence intervals of the 5th (blue), 50th (red), and 95th (blue) percentiles of the simulated data (n = 500 datasets). The black dots correspond to the prediction corrected observed values.

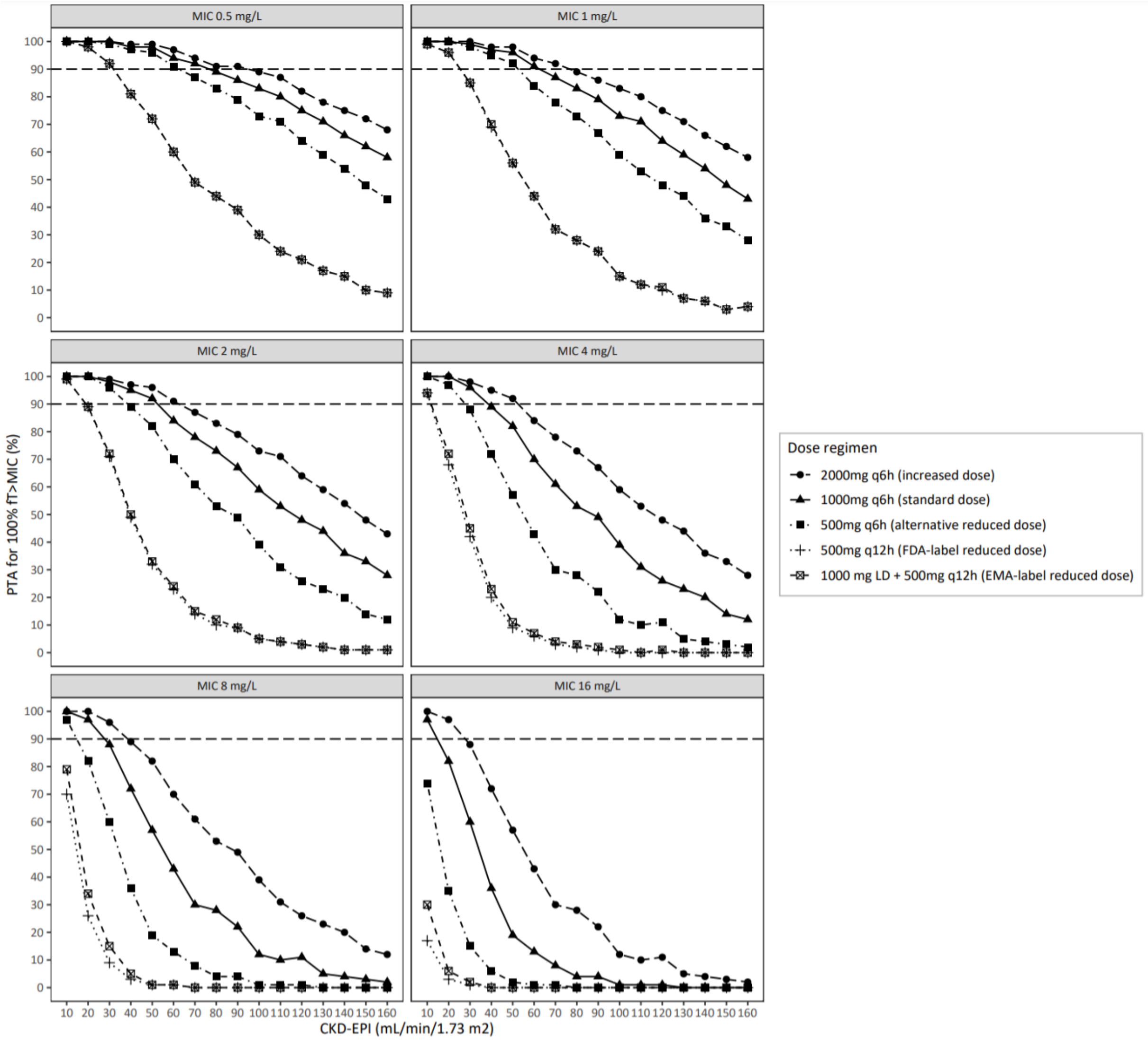

Figure S6. Probability of target attainment (PTA, in %) for the secondary efficacy target of 100% fT>MIC$_{8mg/L}$ on the second day of treatment with IV amoxicillin versus kidney function depicted by CKD-EPI (in ml/min/1.73 m^2) following different dose regimens (n = 16.000 patients per dose regimen). Results are shown for MICs ranging 0.5 – 16 mg/L. Each line represents a different dose regimen. For reference, the dashed line shows 90% PTA as a commonly used minimum target. *PTA* Probability of target attainment.

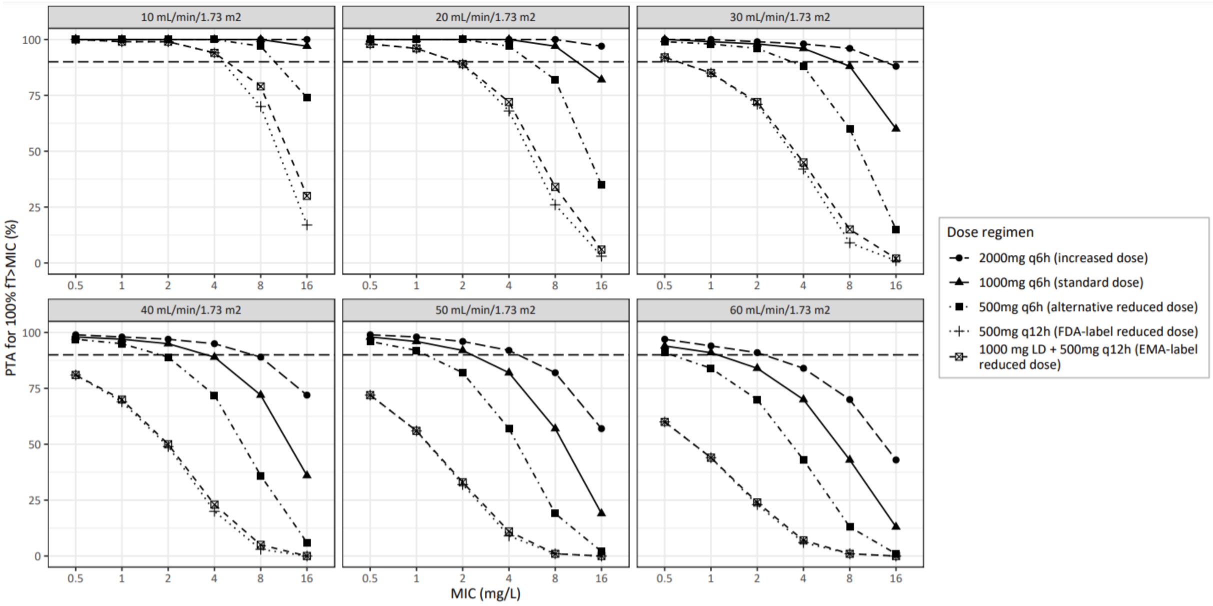

Figure S7. Probability of target attainment (PTA, in %) versus MIC (in mg/L) for different dose regimens (n = 1.000 patients per combination of renal function group and dose regimen) for the secondary efficacy target of 100% *f*T>MIC on the second day of treatment with IV amoxicillin for patients with renal function up to 60 ml/min/1.73 m^2 based on CKD-EPI. For reference, the dashed line shows 90% PTA as a commonly used minimum target. Each line represents a different dose regimen. *MIC* Minimal inhibitory concentration. *PTA* Probability of target attainment.

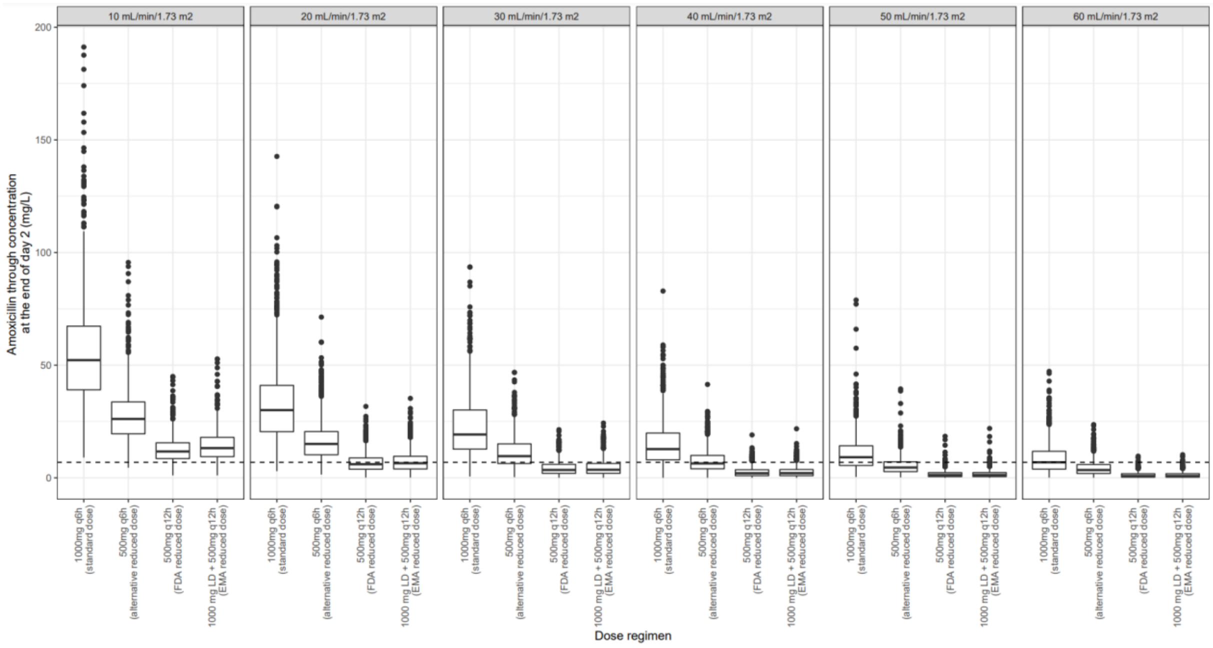

Figure S8. Through amoxicillin concentrations (in mg/L) at the end of day 2 for the standard and reduced dose regimens for different renal function estimates between 10 and 60 ml/min/1.73 m^2 (n = 1000 patients per combination of renal function group and dose regimen). Each panel represents a renal function value. The boxplot shows the median and interquartile range for each dose regimen. The dashed line represents the median amoxicillin through concentration using the standard dose regimen of 1000 mg q6h in patients with a renal function of 60 ml/min/1.73 m^2
